# Supplementary material for: Evaluation of Biofilm Production and Antifungal Susceptibility to Fluconazole in Clinical Isolates of Candida spp. in Both Planktonic and Biofilm Form
Source: Microorganisms. 2024 Jan 12;12(1):153. doi: 10.3390/microorganisms12010153 (PMC10820201; doi:10.3390/microorganisms12010153)
Supplement: Supplementary file 1 [file microorganisms-12-00153-s001.zip › microorganisms-2803075-supplementary.pdf]

microorganisms

MDPI

Supplementary material

Table S1: The following table contains all the information acquired during the work to perform the statistical analysis of the data. It includes the PMIC and MBEC50 values for each individual Candida strain, from which susceptibility could be assessed according to the EUCAST reference breakpoints [15]. For each strain, the respective category of low, medium and high biofilm producer and low, medium and high metabolic activity, evaluated according to tertile analysis, is also given.

N.	Strain	Material	PMIC (mg/l)	MBEC$_{50}$ (mg/l)	PMIC BP	MBEC$_{50}$ BP	CV Category	AB Category
**1**	*Candida albicans*	Blood	0,5	16	S	R	LBF	MMA
**2**	*Candida albicans*	Blood	<=0.12	16	S	R	LBF	MMA
**3**	*Candida albicans*	Blood	0,25	0,25	S	S	LBF	LMA
**4**	*Candida albicans*	Blood	0,25	16	S	R	LBF	LMA
**5**	*Candida albicans*	Blood	0,25	0,25	S	S	LBF	LMA
**6**	*Candida albicans*	Blood	<=0,012	0,03	S	S	HBF	MMA
**7**	*Candida albicans*	Blood	0,25	16	S	R	HBF	HMA
**8**	*Candida albicans*	Blood	0,25	16	S	R	HBF	HMA
**9**	*Candida albicans*	Blood	0,06	0,06	S	S	HBF	HMA
**10**	*Candida albicans*	Blood	0,5	16	S	R	HBF	HMA
**11**	*Candida albicans*	Blood	0,25	16	S	R	HBF	HMA
**12**	*Candida albicans*	Blood	0,5	16	S	R	MBF	MMA
**13**	*Candida albicans*	Blood	0,25	16	S	R	MBF	HMA
**14**	*Candida albicans*	Blood	0,25	16	S	R	HBF	HMA
**15**	*Candida albicans*	Blood	0,25	1	S	S	MBF	HMA
**16**	*Candida albicans*	Blood	0,25	0,5	S	S	HBF	MMA
**17**	*Candida albicans*	Blood	0,25	16	S	R	HBF	MMA
**18**	*Candida albicans*	Blood	0,25	8	S	R	HBF	HMA
**19**	*Candida albicans*	Blood	0,25	16	S	R	MBF	HMA
**20**	*Candida albicans*	Blood	0,25	16	S	R	HBF	MMA
**21**	*Candida albicans*	Blood	0,25	16	S	R	HBF	MMA
**22**	*Candida albicans*	Other material	0,25	16	S	R	MBF	LMA
**23**	*Candida albicans*	Other material	0,5	8	S	R	MBF	LMA
**24**	*Candida albicans*	Other material	0,25	16	S	R	MBF	LMA
**25**	*Candida albicans*	Other material	0,25	16	S	R	HBF	MMA
**26**	*Candida albicans*	Other material	0,25	2	S	S	HBF	LMA
**27**	*Candida albicans*	Other material	0,25	16	S	R	HBF	MMA
**28**	*Candida albicans*	Other material	0,5	16	S	R	MBF	MMA
**29**	*Candida albicans*	Other material	0,25	1	S	S	MBF	LMA

**30**	*Candida albicans*	Other material	0,25	16	S	R	HBF	HMA
**31**	*Candida albicans*	Other material	0,25	8	S	R	HBF	HMA
**32**	*Candida albicans*	Other material	0,25	8	S	R	HBF	MMA
**33**	*Candida albicans*	Other material	0,25	0,25	S	S	MBF	LMA
**34**	*Candida albicans*	Other material	0,25	16	S	R	MBF	MMA
**35**	*Candida albicans*	Other material	0,5	16	S	R	MBF	MMA
**36**	*Candida albicans*	Other material	0,25	16	S	R	MBF	HMA
**37**	*Candida albicans*	Other material	0,25	16	S	R	MBF	HMA
**38**	*Candida albicans*	Other material	0,25	16	S	R	HBF	HMA
**39**	*Candida glabrata*	Blood	16	16	I	R	MBF	MMA
**40**	*Candida glabrata*	Blood	8	8	I	I	LBF	HMA
**41**	*Candida glabrata*	Blood	32	16	R	R	LBF	HMA
**42**	*Candida glabrata*	Blood	16	16	I	R	LBF	HMA
**43**	*Candida glabrata*	Blood	32	16	R	R	LBF	HMA
**44**	*Candida glabrata*	Blood	2	16	I	R	LBF	LMA
**45**	*Candida glabrata*	Blood	0,5	16	I	R	MBF	LMA
**46**	*Candida glabrata*	Blood	16	16	I	R	LBF	LMA
**47**	*Candida glabrata*	Blood	0,5	8	I	I	LBF	LMA
**48**	*Candida glabrata*	Other material	32	16	R	R	LBF	LMA
**49**	*Candida glabrata*	Other material	8	16	I	R	MBF	LMA
**50**	*Candida parapsilosis*	Blood	64	16	R	R	MBF	MMA
**51**	*Candida parapsilosis*	Blood	0,5	8	S	R	LBF	LMA
**52**	*Candida parapsilosis*	Blood	<=0.12	1	S	S	LBF	LMA
**53**	*Candida parapsilosis*	Blood	0,25	16	S	R	MBF	MMA
**54**	*Candida parapsilosis*	Blood	32	16	R	R	MBF	HMA
**55**	*Candida parapsilosis*	Blood	0,5	0,5	S	S	LBF	HMA
**56**	*Candida parapsilosis*	Blood	0,25	8	S	R	LBF	LMA
**57**	*Candida parapsilosis*	Blood	0,5	16	S	R	HBF	HMA
**58**	*Candida parapsilosis*	Blood	32	16	R	R	MBF	MMA
**59**	*Candida parapsilosis*	Blood	32	16	R	R	MBF	HMA
**60**	*Candida parapsilosis*	Blood	0,5	1	S	S	HBF	LMA
**61**	*Candida parapsilosis*	Blood	32	16	R	R	HBF	LMA
**62**	*Candida parapsilosis*	Blood	1	16	S	R	LBF	MMA
**63**	*Candida parapsilosis*	Blood	64	16	R	R	LBF	MMA
**64**	*Candida parapsilosis*	Blood	32	16	R	R	LBF	LMA
**65**	*Candida parapsilosis*	Blood	0,5	16	S	R	MBF	HMA
**66**	*Candida parapsilosis*	Blood	32	16	R	R	LBF	HMA
**67**	*Candida parapsilosis*	Blood	32	16	R	R	LBF	HMA
**68**	*Candida parapsilosis*	Blood	1	8	S	R	MBF	LMA
**69**	*Candida parapsilosis*	Blood	0,25	16	S	R	LBF	LMA

**70**	*Candida parapsilosis*	Blood	0,5	2	S	S	LBF	LMA
**71**	*Candida parapsilosis*	Blood	32	16	R	R	LBF	LMA
**72**	*Candida parapsilosis*	Blood	64	16	R	R	LBF	HMA
**73**	*Candida parapsilosis*	Other material	2	2	S	S	LBF	LMA
**74**	*Candida parapsilosis*	Other material	16	16	S	R	LBF	LMA
**75**	*Candida parapsilosis*	Other material	2	2	S	S	MBF	LMA
**76**	*Candida tropicalis*	Blood	1	16	S	R	MBF	MMA
**77**	*Candida tropicalis*	Blood	0,5	16	S	R	HBF	MMA
**78**	*Candida tropicalis*	Blood	2	2	S	S	HBF	MMA
**79**	*Candida tropicalis*	Blood	16	16	R	R	MBF	HMA
**80**	*Candida tropicalis*	Blood	64	16	R	R	HBF	MMA
**81**	*Candida tropicalis*	Blood	1	16	S	R	HBF	MMA
**82**	*Candida tropicalis*	Other material	1	16	S	R	HBF	MMA
**83**	*Candida tropicalis*	Other material	4	16	R	R	HBF	MMA

PMIC: minimum inhibitory concentration of the planktonic form.
MBEC$_{50}$: minimum concentration of 50% eradication of the biofilm.
PMIC and MBEC$_{50}$ BP: breackpoint as susceptible (S), susceptible, increased exposure (I) and resistant (R), according EUCAST breakpoints **[15].**
CV category: *Candida* strains with low (LBF), moderate (MBF), and high (HBF) biofilm-forming capabilities.
AB category: *Candida* strains with low (LMA), moderate (MMA), and high (HMA) metabolic activities.

Table S2: The increase in resistance by moving from PMIC to MBEC$_{50}$ can be seen in the table. For statistical analysis, each Candida species analyzed was divided according to LBF, MBF, and HBF categories and according to the degree of susceptibility (S, I, R), based on the respective reference breakpoints listed by EUCAST [15].
PMIC: minimum inhibitory concentration of the planktonic form; MBEC$_{50}$: minimum 50% eradication concentration of the biofilm; S: susceptible; I: susceptible, increased exposure; R: resistant.

**Spieces**		**Blood**			**Other materials**			**Blood (%)**			**Other materials (%)**		
Candida albicans		**LBF**	**MBF**	**HBF**	**LBF**	**MBF**	**HBF**	**LBF**	**MBF**	**HBF**	**LBF**	**MBF**	**HBF**
PMIC	**S**	5	4	12	0	10	7	100%	100%	100%	0%	100%	100%
	**R**	0	0	0	0	0	0	0%	0%	0%	0%	0%	0%
MBEC$_{50}$	**S**	2	1	3	0	2	1	40%	25%	25%	0%	20%	14%
	**R**	3	3	9	0	8	6	60%	75%	75%	0%	80%	86%
**Total**		**5**	**4**	**12**	**0**	**10**	**7**	**60%**	**75%**	**75%**	**/**	**80%**	**86%**

**Spieces**	**Blood**	**Other materials**	**Blood (%)**	**Other materials (%)**

microorganisms MDPI

Candida parapsilosis		LBF	MBF	HBF	LBF	MBF	HBF	LBF	MBF	HBF	LBF	MBF	HBF
PMIC	**S**	7	3	2	2	1	0	54%	43%	67%	100%	100%	0%
	**R**	6	4	1	0	0	0	46%	57%	33%	0%	0%	0%
$MBEC_{50}$	**S**	3	0	1	1	1	0	23%	0%	33%	50%	100%	0%
	**R**	10	7	2	1	0	0	77%	100%	67%	50%	0%	0%
**Total**		**13**	**7**	**3**	**2**	**1**	**0**	**31%**	**43%**	**33%**	**50%**	**0%**	**/**

Spieces		Blood			Other materials			Blood (%)			Other materials (%)		
Candida glabrata		**LBF**	**MBF**	**HBF**	**LBF**	**MBF**	**HBF**	**LBF**	**MBF**	**HBF**	**LBF**	**MBF**	**HBF**
PMIC	**S**	0	0	0	0	0	0	0%	0%	0%	0%	0%	0%
	**I**	5	2	0	0	1	0	71%	100%	0%	0%	100%	0%
	**R**	2	0	0	1	0	0	29%	0%	0%	100%	0%	0%
$MBEC_{50}$	**S**	0	0	0	0	0	0	0%	0%	0%	0%	0%	0%
	**I**	2	0	0	0	0	0	29%	0%	0%	0%	0%	0%
	**R**	5	2	0	1	1	0	71%	100%	0%	100%	100%	0%
**Total**		**7**	**2**	**0**	**1**	**1**	**0**	**43%**	**100%**	**/**	**0%**	**100%**	**/**

Spieces		Blood			Other materials			Blood (%)			Other materials (%)		
Candida tropicalis		**LBF**	**MBF**	**HBF**	**LBF**	**MBF**	**HBF**	**LBF**	**MBF**	**HBF**	**LBF**	**MBF**	**HBF**
PMIC	**S**	0	1	3	0	0	1	0%	50%	75%	0%	0%	50%
	**R**	0	1	1	0	0	1	0%	50%	25%	0%	0%	50%
$MBEC_{50}$	**S**	0	0	1	0	0	0	0%	0%	25%	0%	0%	0%
	**R**	0	2	3	0	0	2	0%	100%	75%	0%	0%	100%
**Total**		**0**	**2**	**4**	**0**	**0**	**2**	**/**	**50%**	**50%**	**/**	**/**	**50%**
